# The novel thiosemicarbazone, di-2-pyridylketone 4-cyclohexyl-4-methyl-3-thiosemicarbazone (DpC), inhibits neuroblastoma growth in vitro and in vivo via multiple mechanisms

**DOI:** 10.1186/s13045-016-0330-x

**Published:** 2016-09-27

**Authors:** Zhu-Ling Guo, Des R. Richardson, Danuta S. Kalinowski, Zaklina Kovacevic, Kian Cheng Tan-Un, Godfrey Chi-Fung Chan

**Affiliations:** 1 Department of Stomatology, Affiliated Hospital of Hainan Medical University, Hainan, People’s Republic of China; 2 School of Stomatology, Hainan Medical University, Hainan, People’s Republic of China; 3 Department of Paediatrics & Adolescent Medicine, Queen Mary Hospital, The University of Hong Kong, Hong Kong, SAR China; 4 Molecular Pharmacology and Pathology Program, Department of Pathology, University of Sydney, Sydney, New South Wales Australia; 5 School of Professional and Continuing Education, The University of Hong Kong, Hong Kong, SAR People’s Republic of China

**Keywords:** Thiosemicarbazone, di-2-pyridylketone 4,4-dimethyl-3-thiosemicarbazone (Dp44mT), molecular pharmacology, cancer treatment, neuroblastoma

## Abstract

**Background:**

Neuroblastoma is a relatively common and highly belligerent childhood tumor with poor prognosis by current therapeutic approaches. A novel anti-cancer agent of the di-2-pyridylketone thiosemicarbazone series, namely di-2-pyridylketone 4,4-dimethyl-3-thiosemicarbazone (Dp44mT), demonstrates promising anti-tumor activity. Recently, a second-generation analogue, namely di-2-pyridylketone 4-cyclohexyl-4-methyl-3-thiosemicarbazone (DpC), has entered multi-center clinical trials for the treatment of advanced and resistant tumors. The current aim was to examine if these novel agents were effective against aggressive neuroblastoma in vitro and in vivo and to assess their mechanism of action.

**Methods:**

Neuroblastoma cancer cells as well as immortalized normal cells were used to assess the efficacy and selectivity of DpC in vitro. An orthotopic SK-N-LP/Luciferase xenograft model was used in nude mice to assess the efficacy of DpC in vivo. Apoptosis in tumors was confirmed by Annexin V/PI flow cytometry and H&E staining.

**Results:**

DpC demonstrated more potent cytotoxicity than Dp44mT against neuroblastoma cells in a dose- and time-dependent manner. DpC significantly increased levels of phosphorylated JNK, neuroglobin, cytoglobin, and cleaved caspase 3 and 9, while decreasing IkBα levels in vitro. The contribution of JNK, NF-ĸB, and caspase signaling/activity to the anti-tumor activity of DpC was verified by selective inhibitors of these pathways. After 3 weeks of treatment, tumor growth in mice was significantly (*p* < 0.05) reduced by DpC (4 mg/kg/day) given intravenously and the agent was well tolerated. Xenograft tissues showed significantly higher expression of neuroglobin, cytoglobin, caspase 3, and tumor necrosis factor-α (TNFα) levels and a slight decrease in interleukin-10 (IL-10).

**Conclusions:**

DpC was found to be highly potent against neuroblastoma, demonstrating its potential as a novel therapeutic for this disease. The ability of DpC to increase TNFα in tumors could also promote the endogenous immune response to mediate enhanced cancer cell apoptosis.

**Electronic supplementary material:**

The online version of this article (doi:10.1186/s13045-016-0330-x) contains supplementary material, which is available to authorized users.

## Background

**Fig. 1:**
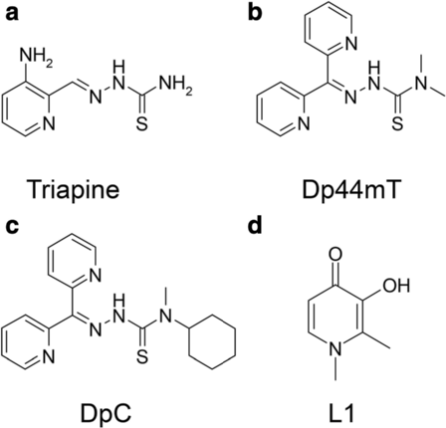
Line drawings of the structures of **a** Triapine, **b** Dp44mT, **c** DpC, and **d** L1

The thiosemicarbazone, 3-aminopyridine-2-carboxaldehyde thiosemicarbazone (3-AP/Triapine; Fig. 1a), has undergone over 20 Phase I and Phase II clinical trials [1]. However, the side effects of this agent have hindered its clinical application [2]. Based on comprehensive structure-activity relationship studies [3–6], our team developed alternative agents of the di-2-pyridylketone thiosemicarbazone (DpT) class [7, 8] to overcome the disadvantages of Triapine. The DpT analogues bind iron and copper to generate oxidative stress in cancer cells, which induces lysosomal membrane permeabilization [9–13] and results in these agents overcoming P-glycoprotein-mediated drug resistance [10, 12, 13]. These compounds also induce apoptotic and autophagic pathways [8, 9, 14] and inhibit tumorigenic processes [15–21].

Due to its high efficacy and selectivity, di-2-pyridylketone 4,4-dimethyl-3-thiosemicarbazone (Dp44mT; Fig. 1b) was chosen as the first lead DpT analogue [7, 8] with its marked activity being confirmed by others [22–24]. Importantly, this agent has been demonstrated to upregulate the potent metastasis suppressor, N-myc downstream-regulated gene-1 (NDRG1) [25], which inhibits the epithelial to mesenchymal transition [15] and results in suppression of oncogenic signaling, tumor cell migration [15–21], and metastasis in vivo [23].

However, due to cardiac fibrosis at high, non-optimal Dp44mT doses [7], a second generation of DpT analogues was synthesized, resulting in a new lead agent, namely di-2-pyridylketone 4-cyclohexyl-4-methyl-3-thiosemicarbazone (DpC; Fig. 1c), that demonstrates high tolerability [26, 27]. In fact, early in 2016, DpC entered multi-center clinical trials for treating advanced tumors (NCT02688101), which again supports its selectivity, tolerability, and favorable pharmacological properties [28]. Significantly, while DpC shares structural similarities to Dp44mT (cf*.* Fig. 1b, c), it demonstrates a series of important advantages. These include the following: (1) DpC, unlike Dp44mT, does not induce cardiac fibrosis even when administered at markedly higher doses [26, 27]; (2) Unlike Dp44mT and Triapine, DpC does not induce oxyhemoglobin oxidation in vivo [2]; (3) DpC exhibits greater activity than Dp44mT in vivo against an aggressive human pancreatic tumor xenograft [26]; (4) DpC demonstrated pronounced in vivo activity after oral and intravenous administration [27], while Dp44mT was not tolerated orally [29]; and (**5**) while both Dp44mT and DpC display appropriate pharmacokinetics, the markedly greater half-life of DpC (*t*_1/2_ = 10.7 h for DpC vs. 1.7 h for Dp44mT) further underlines its potential [30].

Considering the marked anti-tumor activity of DpC and its favorable pharmacology and safety profile, it is notable that it has not yet been examined for the treatment of belligerent neuroblastoma. While the outcomes of many childhood cancers have improved, advanced neuroblastoma has a dismal prognosis [31–35]. However, it is notable that neuroblastoma is sensitive to iron chelation with standard chelators, such as deferiprone (L1; Fig. 1d) [36] and desferrioxamine (DFO) alone, or in combination with cytotoxic chemotherapy [37–41]. This is despite the fact that DFO and L1 show only low to moderate anti-tumor activity [36], which is far less marked than Dp44mT or DpC [7, 8, 26, 27].

In view of the pronounced anti-tumor activity of Dp44mT and DpC and the sensitivity of neuroblastoma to iron chelation, this study assessed the activity of these agents against neuroblastoma in vitro and in vivo with the aim to investigate the anti-tumor mechanisms involved. The results demonstrate that DpC shows marked and selective anti-tumor activity, which could be useful for the treatment of neuroblastoma.

## Methods

### Cell lines

The human neuroblastoma cell lines, SK-N-LP (provided by Dr. Nai-Kong Cheung, Memorial Sloan Kettering Cancer Center, New York, NY, USA), BE(2)C, SK-N-AS, and SH-SY5Y were purchased from the American Type Culture Collection (ATCC; Manassas, VA, USA). The following non-tumorigenic, immortalized normal cell lines were also used: the human kidney cell line (HK2; ATCC), human hepatocyte cell line (MIHA; ATCC); the human bone marrow-derived Tert-immortalized mesenchymal stem cell line (MSC; from Prof. D. Campana, St. Jude Children’s Research Hospital, Memphis, Tennessee, USA); and rat cardiomyocyte cell line (H9C2; from Prof. M. Yang, Nanfang University, Guangzhou, Guangdong, China).

All neuroblastoma cell lines and the HK2 and MIHA cells were maintained in Dulbecco’s modified Eagle medium—high glucose (Invitrogen, Grand Island, New York, NY, USA), supplemented with 100 U/mL penicillin, 100 mg/mL streptomycin (Invitrogen), and 10 % heat-inactivated fetal bovine serum (Hyclone, Logan, USA). For the SK-N-LP cell line expressing Luciferase, G418 (1000 μg/mL; Roche, Mannheim, Germany), was added to the media to maintain selective pressure. The human MSC line was cultured using Dulbecco’s modified Eagle medium—low glucose (Invitrogen). All cells were kept under standard culture conditions at 37 °C in a humidified 5 % CO_2_ atmosphere with the culture medium being renewed every other day. The H9C2 cell type was cultured in culture vessels pre-coated with 0.02 % gelatin (Difco, Fisher Scientific, Suwanee, GA, USA) and 5 μg/mL fibronectin (Sigma-Aldrich) solution at 37 °C in a humidified 5 % CO_2_ incubator, maintained in Claycomb media (Sigma-Aldrich) supplemented with 10 % fetal bovine serum (Sigma-Aldrich), 0.1 mM norepinephrine (Sigma-Aldrich), 2 mM l-glutamine (Invitrogen), and penicillin/streptomycin (100 U/mL and 100 μg/mL, respectively; Invitrogen).

### Chemical agents

Both DpC and Dp44mT were synthesized and characterized as described previously [27, 42]. Both DpC (5 mg) and Dp44mT (5 mg) were freshly dissolved in 1 mL DMSO to generate a 5 mg/mL solution and then diluted in media for use. On the other hand, L1 (Apotex Inc., Toronto, ONT, Canada) was dissolved in doubly distilled H_2_O. The cell lines described above were incubated with either DpC, Dp44mT, or L1 at concentrations of 2.5, 25, or 250 μM for 0, 12, 24, 48, or 72 h/37 °C.

### XTT proliferation assay

Cells were seeded in 96-well plates (approximately 4000 cells/well). After overnight culture, cells were incubated with either control medium or medium containing DpC, Dp44mT, or L1. Cellular proliferation was then assessed after incubations of 24, 48, or 72 h/37 °C using the XTT kit (Roche). The optical density was measured using a microplate reader at a wavelength of 450 nm. Cellular proliferation was demonstrated to be directly correlated to cell number, as shown for the related MTT assay [4].

In studies using cell signaling pathway inhibitors, cells were pre-incubated for 2 h/37 °C with 5 μM of the ERK/MAPK inhibitor, PD98059 (Sigma-Aldrich), 5 μM of the p38 MAPK inhibitor, SB203580 (Sigma-Aldrich), 5 μM of the JNK/MAPK inhibitor, SP600125 (Sigma-Aldrich), 15 μg/mL of the NF-kB inhibitor, CAPE (Sigma-Aldrich), and 10 μM of the pan-caspase inhibitor, Z-VAD(ome)-FMK (Calbiochem, Darmstad, Germany). The viability of neuroblastoma cells after a 24 h/37 °C incubation with DpC, Dp44mT, or L1 in the presence or absence of the inhibitors was examined using the XTT kit, as described above.

### Flow cytometry

Mouse tissues from the tumor, heart, lung, spleen, liver, kidney, and brain were weighed, homogenized, and filtered using a 70-μm cell strainer on ice. Suspensions containing approximately 5 × 10^4^ single cells were rapidly prepared (within 1 h) to perform flow cytometry. Cell lines treated with control media alone or this media in the presence or absence of 1.4 % DMSO or DpC, Dp44mT, or L1 (all at 25 μM) were also examined using this technique. The cells that were Annexin V+/PI−, Annexin V+/PI+, and Annexin V−/PI+ were divided as either the early apoptosis group, late apoptosis group, or necrotic group, respectively. The levels of caspase 3 expression induced by DpC were detected using the FITC Active Caspase-3 Apoptosis Kit (BD Biosciences, San Diego, USA). Antibodies against Ngb and Cygb (Abcam, Cambridge, UK) were kindly provided by Dr. Tan-Un (School of Professional and Continuing Education, The University of Hong Kong, Hong Kong, People’s Republic of China). Data were analyzed by using Flow Jo 8.8.2.

### Effect of DpC on the growth of an orthotopic neuroblastoma in nude mice

Four-week-old male nude mice (BALB/c nu/nu) were acquired from the Laboratory Animal Unit of the University of Hong Kong with the approval of the Hong Kong Department of Health and also the Committee for the Use of Live Animals in Teaching and Research at the University of Hong Kong (CULATR 3131-13). Mice were routinely anesthetized and disinfected prior to the abdominal operation. Using a surgical operation microscope, 2 × 10^5^ SK-N-LP/Luciferase cells diluted in 50 % Matrigel® (BD Biosciences) were administered directly into the fat pad of the left-side adrenal gland of the mouse. By intraperitoneal injection of luciferin (Invitrogen), the condition of the xenograft (with a volume of <4000 mm^3^) was monitored via a Xenogen In Vivo Imaging System (Xenogen, CA, USA)*.* The region of interest (ROI) was generated automatically and its value was normalized under the luminescence interval of 17 × 10^4^ to 2.7 × 10^5^.

Two weeks post-neuroblastoma transplantation, the mice were divided into two groups according to the tumor ROI value. The mice were then treated with either DpC (4 mg/kg) or the vehicle control (i.e., DMSO/PBS) administered via the tail vein daily for 3 weeks. Mouse body weight and temperature were recorded daily and weight loss monitored to ensure that it did not exceed 10 % at any time (due to ethics requirements at Hong Kong University). Then, the mice were sacrificed by an overdose of pentobarbital. Tissues from the tumor, heart, lung, spleen, liver, kidney, and brain were harvested for ex vivo experiments. The length, width, and height of the tumors were measured using digital calipers to calculate the final xenograft volumes, using the formula: 4/3 × π (length × width × height)/8.

### Histopathology

Approximately 0.5–1 cm^3^ of mouse tissue taken from the tumor, heart, lung, spleen, liver, kidney, and brain was resected and immediately immersed in 4 % paraformaldehyde for overnight fixation. The paraffin-embedded blocks were sectioned and mounted on slides using 4-μm slices. Then, H&E staining was performed to evaluate histopathology. Pictures were taken using a bright-field microscope at ×400 magnification.

### Western blotting

SK-N-LP cells were lysed directly with radioimmunoprecipitation assay (RIPA) buffer for 2 h/4 °C with constant agitation. Lysates were clarified by centrifugation for 20 min/12,000 rpm/4 °C and the protein concentrations were quantified using the Bio-Rad Protein Assay Kit (Bio-Rad, Hercules, CA, USA). SDS-PAGE and western blotting were performed using standard techniques [43].

The Spectra Multi-Color Protein Ladder (Thermo Fisher Scientific Inc., New York, NY, USA) was used as molecular weight markers in gel electrophoresis and western blotting experiments. The primary rabbit polyclonal antibodies of phosphorylated and total ERK, P38 and JNK, caspase 3 (Cell Signaling Technology, Danvers, MA, USA), neuroglobin, cytoglobin, IkBα (Santa Cruz Biotechnology, Dallas, TX, USA), as well as mouse monoclonal antibody against cleaved caspase 9 (Cell Signaling Technology) were used at a dilution of 1:1000 in PBS-Tween 20 (Bio-Rad) containing 5 % bovine serum albumin (Sigma-Aldrich).

As an appropriate protein-loading control, a primary β-actin (CST 4967) antibody at a dilution of 1:8000 was utilized. Subsequently, a secondary anti-rabbit antibody at a dilution of 1:4000 was used and the resulting immune complex visualized by enhanced chemiluminescence (Pierce, Chicago, IL, USA). The density of the protein bands was calculated using Quantity One software (Bio-Rad).

### ELISA assay

Approximately 1.5 g of tumor tissue was homogenized, filtered, and centrifuged at 4 °C. Concentrations of TNFα, IFNγ, and IL-10 in the collected supernatant (approximately 750 μL) were measured using a mouse ELISA kit (Ebioscience, San Diego, CA, USA) according to the manufacturer’s instructions. The optical density was measured using a microplate reader at a wavelength of 450 nm with correction at 570 nm.

### Statistical analysis

Statistical analysis was performed using the GraphPad Prism Software Package (v.5, GraphPad Software, San Diego, USA). Differences between groups were analyzed using the unpaired, two-tailed Student’s *t* test. Mice survival analysis was performed by generating Kaplan-Meier survival curves. All data are presented as the mean ± SEM of at least three experiments. It was considered that *p* values less than 0.05 were statistically significant.

## Results

### In vitro cytotoxic activity of DpC and Dp44mT relative to the commercially available chelator, L1, against a panel of non-tumorigenic, immortalized cell lines and the neuroblastoma cell line, SK-N-LP

**Fig. 2:**
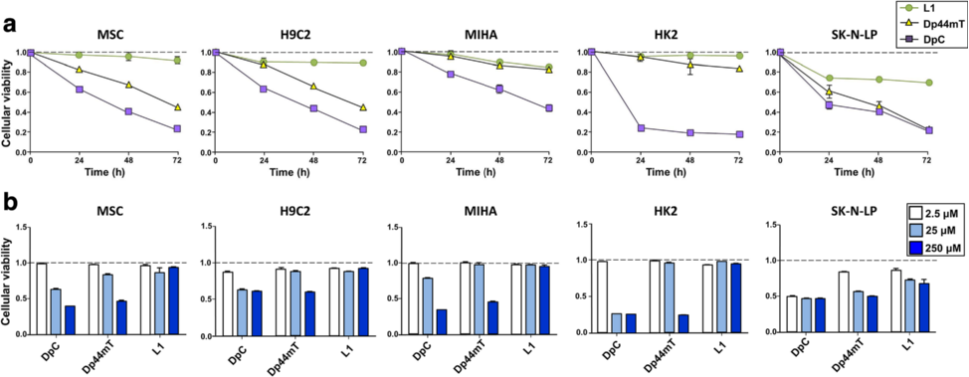
DpC has more potent cytotoxic effects than Dp44mT and L1 on a neoplastic, neuroblastoma cell line, SK-N-LP, than a panel of non-tumorigenic, immortalized cells (i.e., MSC, HSC2, MIHI, HK2). In vitro cellular proliferation was assessed by the XTT assay comparing a panel of non-tumorigenic, immortalized cells and a neuroblastoma cell line incubated with either DpC, Dp44mT, or L1. **a** The effect of DpC, Dp44mT, or L1 (25 μM) on proliferation and viability as a function of time relative to control media alone (24–72 h/37 °C). **b** The effect of DpC, Dp44mT, or L1 at concentrations of 2.5, 25, and 250 μM on proliferation and viability relative to control medium alone after a 24 h/37 °C incubation. Data are presented as mean ± SEM (*n* = 3)

Initial studies examined the selective anti-proliferative activity of DpC and Dp44mT relative to the well-characterized and commercially available chelator, L1, against a panel of non-tumorigenic, immortalized cells compared to a neuroblastoma cell line (Fig. 2). As determined by the XTT assay, the agents DpC and Dp44mT at a relatively high concentration of 25 μM, inhibited the proliferation and viability of the following immortalized, non-tumorigenic cell lines as a function of time (24–72 h/37 °C): human bone marrow-derived, Tert-immortalized mesenchymal stem cells (MSC), rat cardiomyocytes (H9C2), a human hepatocyte cell line (MIHA) and human kidney cells (HK2), as well as the neoplastic, neuroblastoma cell line, SK-N-LP (Fig. 2a). On the other hand, L1 (25 μM) was consistently less effective than Dp44mT in MSC and H9C2 cells, while being markedly less active than DpC against all cell types. Notably, L1 demonstrated low activity against the immortalized, non-tumorigenic cell lines, especially MSC and HK2 cells, but demonstrated relatively higher activity against neoplastic SK-N-LP cells (Fig. 2a).

At 25 μM, Dp44mT demonstrated significantly (*p* < 0.001–0.01) less anti-proliferative activity than DpC in the panel of non-tumorigenic, immortalized cells (Fig. 2a). In fact, Dp44mT showed similar anti-proliferative efficacy to L1 when incubated with the non-tumorigenic, immortalized MIHA and HK2 cells, but was significantly (*p* < 0.001–0.01) more effective at inhibiting proliferation than L1 in non-tumorigenic, MSC, and H9C2 cells after 48 or 72 h. Against the neoplastic SK-N-LP cell-type, Dp44mT and particularly DpC showed significantly (*p* < 0.001–0.05) greater anti-proliferative activity than L1 after incubations of 24–72 h (Fig. 2a).

Examining the efficacy of the agents after a 24 h/37 °C incubation on the panel of non-tumorigenic, immortalized cell lines (i.e., MSC, H9C2, MIHA, and HK2) as a function of concentration (2.5, 25, or 250 μM), it was notable that Dp44mT and DpC showed generally similar anti-proliferative activity (Fig. 2b). However, at 25 μM, DpC demonstrated significantly (*p* < 0.001–0.05) greater efficacy than Dp44mT in all non-tumorigenic cell-types. Further, at a concentration of 250 μM, these thiosemicarbazones were significantly (*p* < 0.001–0.05) more potent than L1 against all cell lines examined (Fig. 2b). Assessing the neoplastic SK-N-LP cell line, it was evident that L1 demonstrated significantly (*p* < 0.05) greater activity at 25 and 250 μM than that observed against the non-tumorigenic, immortalized cell-types. In addition, both Dp44mT, and particularly DpC, were significantly (*p* < 0.01–0.05) more effective against SK-N-LP cells than L1 at 25 and 250 μM (Fig. 2b).

Regarding the selective anti-proliferative activity of these agents in neoplastic cells over non-tumorigenic, immortalized cells, which was observed for Dp44mT and DpC previously [8, 27, 42], it was notable that DpC, Dp44mT, and L1 at 2.5 μM showed no pronounced anti-proliferative activity against the non-tumorigenic, immortalized cell lines (i.e., MSC, H9C2, MIHA, and HK2), but was significantly (*p* < 0.001–0.05) more effective at inhibiting neoplastic SK-N-LP neuroblastoma cells (Fig. 2b). In these tumor cells, the activity of DpC at 2.5 μM was markedly and significantly (*p* < 0.001) greater than either Dp44mT or L1, demonstrating its greater potency (Fig. 2b). As the concentration of Dp44mT or DpC was increased to 25 or 250 μM, the selectivity against the neoplastic SK-N-LP cells relative to the non-tumorigenic immortalized cells was reduced or lost (Fig. 2b). This observation indicates the existence of a “therapeutic window” against the neoplastic cells, which, when exceeded, results in non-tumorigenic cell cytotoxicity [44]. Similar therapeutic responses between non-tumorigenic and neoplastic cells are also generally observed for other types of cytotoxic chemotherapeutics, demonstrating the importance of an optimal dose [44]. Table 1 shows the IC_50_ values calculated for the data shown in Fig. 2b.

**Table 1:** IC_50_ values (μM) for DpC, Dp44mT, and L1 in MSC, H9C2, MIHA, HK2, and SK-N-LP cells

	DpC	Dp44mT	L1
MSC	145.23 ± 13.58	227.32 ± 5.04	>250
H9C2	>250	>250	>250
MIHA	165.73 ± 5.81	227.69 ± 9.93	>250
HK2	17.27 ± 0.19	167.08 ± 0.94	>250
SK-N-LP	<2.5	249.39 ± 51.03	>250

The MSC, H292, MIHA, HK2, and SK-N-LP cells were incubated for 24 h/37 °C with either control, DpC, Dp44mT, or L1 (see “Methods”). The IC_50_ values (μM) are presented as mean ± standard deviation (*n* = 3)

### DpC induces greater apoptosis in neuroblastoma cells than either Dp44mT or L1

**Fig. 3:**
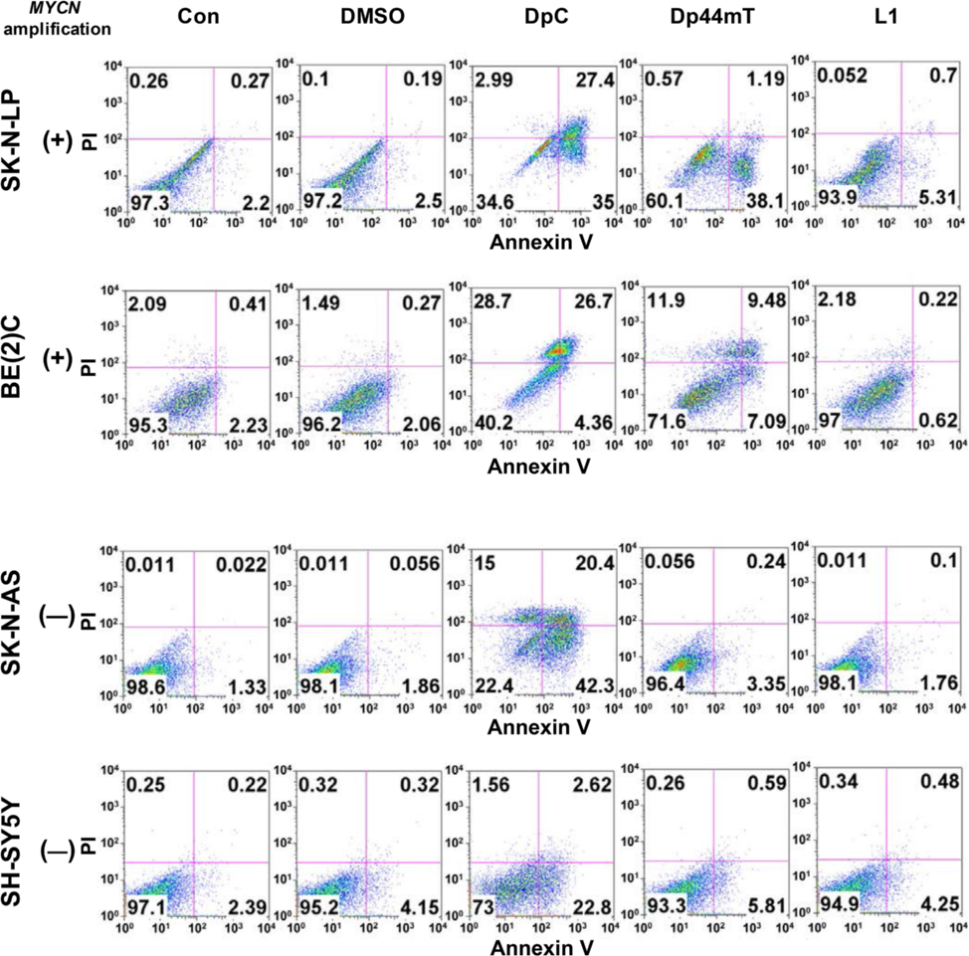
The effects of Dp44mT, DpC, and L1 on apoptosis in four neuroblastoma cell lines, namely SK-N-LP, BE(2)C, SK-N-AS, and SH-SY5Y, as judged by flow cytometric analysis. Cells were incubated with either control media alone (*Con*), control media containing DMSO (*DMSO*), DpC, Dp44mT, or L1 (25 μM) for 24 h/37 °C. Apoptosis was examined using Annexin V/PI staining by flow cytometry. Cells that were Annexin V+/PI+ (*top right quadrant*) were defined as being in late apoptosis, while cells being Annexin V+/PI– (bottom right quadrant) were considered to be in early apoptosis. Cells that were Annexin V–/PI+ (*top left quadrant*) were considered to be necrotic and those that were negative for both (bottom left quadrant) were viable cells. The values shown represent the percentage of cells in each quadrant. Results show a typical experiment of three performed

Considering the results in Fig. 2a, b demonstrating the anti-proliferative activity of these agents, studies then investigated the effect of a 24 h/37 °C incubation with either: control medium (Con), control medium containing the solvent DMSO (final [DMSO] 1.4 % *v*/*v*), DpC, Dp44mT, or L1 (25 μM) on the apoptosis of a panel of four neuroblastoma cell lines via examining Annexin V/PI staining by flow cytometry (Fig. 3). The cells were grouped into (A) live cells (bottom left quadrant; Fig. 3), (B) necrotic cells (top left quadrant; Fig. 3), (C) those undergoing early apoptosis (bottom right quadrant; Fig. 3), or (D) late apoptosis (top right quadrant; Fig. 3) based on the Annexin V/PI staining and expressed as a percentage of total cells. Notably, quantitation of cells in each of these groups is presented in the relevant quadrant in Fig. 3.

Furthermore, these studies also assessed the ability of these agents to induce apoptosis in neuroblastoma cells with and without *MYCN* overexpression. This was important as *MYCN* amplification and its overexpression in neuroblastoma tumors is one of the most powerful predictors of poor prognosis in neuroblastoma [45–47]. In the studies in Fig. 3, SK-N-LP and BE(2)C neuroblastoma cells, which possess *MYCN* amplification, were compared to the SK-N-AS and SH-SY5Y neuroblastoma cell lines, which do not possess *MYCN* amplification [48].

Most cells after incubation with control medium or this medium containing DMSO were viable (95.2–98.6 %; Fig. 3), with only a very low percentage of cells in late-stage apoptosis (0.022–0.41 %; Fig. 3). Examining early apoptosis, DpC had similar effects to Dp44mT in the two *MYCN* amplified neuroblastoma cell lines, including SK-N-LP (35 % of DpC- vs. 38.1 % of Dp44mT-treated cells in early apoptosis) and BE(2)C 4.36 % of DpC- vs. 7.09 % of Dp44mT-treated cells in early apoptosis). However, when assessing late-stage apoptosis, DpC was more effective than Dp44mT in SK-N-LP (27.4 % of DpC- vs. 1.19 % of Dp44mT-treated cells) and BE(2)C cells (26.7 % of DpC- vs. 9.48 % of Dp44mT-treated cells; Fig. 3), suggesting a more potent mechanism of action for DpC. Further, DpC displayed greater activity than Dp44mT in the remaining neuroblastoma cell lines without *MYCN* amplification (i.e., SK-N-AS and SH-SY5Y) in terms of both early and late apoptosis. Hence, DpC and Dp44mT generally demonstrated greater activity in neuroblastoma cells with *MYCN* amplification when compared to those without this alteration. Compared to DpC and Dp44mT, L1 had only very modest anti-neuroblastoma activity in terms of inducing apoptosis, with only 0.1–5.31 % of cells in early or late apoptosis (Fig. 3). Overall, DpC was the most active agent in inducing apoptosis in the 4 neuroblastoma cell lines and importantly demonstrated marked activity irrespective of *MYCN* amplification (Fig. 3).

### Growth inhibition of orthotopic neuroblastoma in a nude mouse model after DpC treatment

**Fig. 4:**
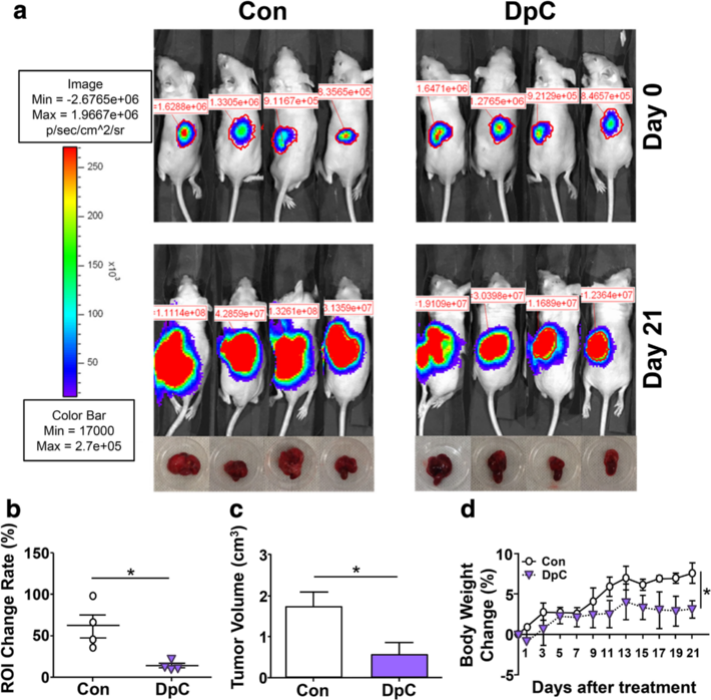
Treatment with intravenous (tail vein) DpC (4 mg/kg/day) over 3 weeks significantly reduces orthotopic neuroblastoma tumor growth (in adrenal gland) in vivo using SK-N-LP/Luciferase xenografts in nude mice. **a** *Upper panel* mice are shown before administration via the tail vein of the vehicle control (*Con*) or DpC (*Day 0*), while the lower panel represents mice treated intravenously (tail vein) with either DpC (4 mg/kg/day) or the vehicle control daily for 3 weeks (i.e., *Day 21*). **b** DpC-treated tumors had significantly (*p* < 0.05) lower region of interest (*ROI*) change rates when compared to vehicle control tumors. **c** Neuroblastoma xenografts significantly decreased in volume after the DpC treatment in (**a**). **d** Body weight change (%) of mice treated with the control (vehicle) or DpC (4 mg/kg/day) over 3 weeks. *p < 0.05 by an unpaired two-tailed *t* test. The results are presented as mean ± SEM (*n* = 4)

Considering the marked activity of DpC against neuroblastoma cells in vitro (Figs. 2 and 3), studies then progressed to assess its selective anti-tumor activity in vivo using nude mice bearing an orthotopic neuroblastoma in the fat pad of the left adrenal gland (Fig. 4a–c). This model has been used previously to assess the anti-neuroblastoma activity of other potential chemotherapeutics [49, 50]. In these studies, groups of mice (*n* = 4) underwent daily intravenous injection with the vehicle control or DpC (4 mg/kg) for 3 weeks and tolerated this intensive treatment routine well. At the end of the study, the mice were then sacrificed for tumor size comparison. Notably, the 3-week treatment period was the maximum that could be utilized due to the rapid growth of the tumor in the control group, which necessitates euthanasia to satisfy the pre-set, local ethical requirements. Post-mortem neuroblastoma xenografts showed a significant (*p* < 0.05) decrease of the in vivo tumor imaging ROI value (Fig. 4b) and also the tumor volume in the DpC-treated mice relative to the vehicle-treated control (Fig. 4c).

No surface temperature fluctuations of the mice were found post-DpC administration during the entire treatment period (data not shown). Although mouse body weights in the DpC-treated group did not show a distinct decline, their weight gain within the 3-week treatment period showed a slight, but significant (*p* < 0.05) decrease relative to that of the control group (Fig. 4d). The slight reduction in weight gain in mice treated with DpC is in contrast to previous studies, where similar treatment regimens did not significantly (*p* > 0.05) affect animal weight [26]. The reason for the slight difference between these investigations could be the more intensive treatment regime in the current study, where the animals were given DpC every day for 3 weeks. This is in contrast to the previous study, where the mouse was treated for 5 days/week with 2 days of rest before undergoing the next cycle of treatment [26].

### Evaluation of the therapeutic effect of DpC in the orthotopic neuroblastoma mouse model

**Fig. 5:**
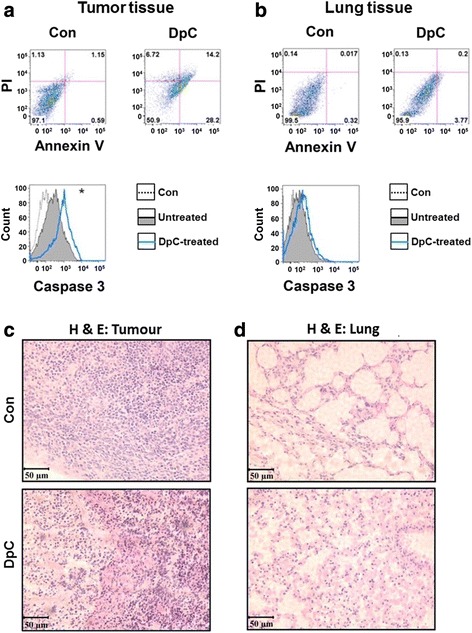
**a** Flow cytometric analysis demonstrates that treatment of nude mice bearing an orthotopic neuroblastoma xenograft with intravenous DpC (4 mg/kg/day) administered via the tail vein over 3 weeks increases Annexin V and caspase 3 expression in the tumor, but not in **b** the lung. *p < 0.05 by an unpaired two-tailed Student’s *t* test in three nude mice after 21 days of DpC treatment. **c**, **d** Histopathological assessment (hematoxylin and eosin, *H&E*) of the tumor (**c**) and lung (**d**) after 3 weeks of intravenous treatment of mice with either the vehicle control or DpC (4 mg/kg/day). **c** H&E staining demonstrating a decrease in tumor cell infiltration after DpC treatment relative to the vehicle control and **d** evidence of exudative inflammation could be observed in lung tissue of nude mice following treatment with DpC relative to the control. The results in (**a**) and (**b**) are typical experiments from three performed. The results in (**c**) and (**d**) are typical photographs from sections of tissue. *Scale bar* on H&E photographs, 50 μm

In DpC-treated mice relative to the control, significantly (*p* < 0.05) higher levels of Annexin V (+)/PI (+) cells and caspase 3 were demonstrated in tumor tissues post-mortem (Fig. 5a). Indeed, assessment of the percentage of live tumor cells in the controls (97.1 %) was far greater than in the DpC-treated group (50.9 %), with a marked increase in the percentage of tumor cells in early- or late-stage apoptosis after DpC treatment (28.2 and 14.2 %, respectively) relative to the control group (0.59 and 1.15 %, respectively). These results clearly demonstrated the marked anti-neuroblastoma activity of DpC.

In contrast, upon examining normal tissues, e.g., the lung (Fig. 5b), no evidence of significantly increased Annexin V (+)/PI (+) cells or caspase 3 was observed. Flow cytometric examination of the percentage of viable cells in the lungs of the controls (99.5 %) was similar (*p* > 0.05) to the DpC-treated group (95.9 %), there being a small increase in the percentage of lung cells in late-stage apoptosis after DpC treatment (0.2 %) relative to the control group (0.017 %; Fig. 5b). Similarly, no marked alterations in these parameters were also observed in a variety of other normal tissues (i.e., spleen, heart, kidney, and brain; data not shown). However, significant neuroblastoma xenograft regression was confirmed by H&E staining (Fig. 5c). Histopathological examination of H&E-stained sections of the lungs suggested some evidence of exudative inflammation (Fig. 5d), while cellular morphology remained normal in the spleen, heart, kidney, and brain (data not shown). The alterations observed in the lung with DpC treatment were not reported in a previous study with this agent in another in vivo tumor model [26]. Again, this may indicate that the more intensive treatment regimen used in the current experiments was outside the therapeutic window and led to some limited adverse effects on the lungs.

### Mechanism of the anti-neuroblastoma activity of DpC and Dp44mT

Considering the marked anti-neuroblastoma activity of DpC and Dp44mT in vitro (Figs. 2 and 3) and the in vivo efficacy of DpC in the mouse neuroblastoma model (Fig. 4), studies then examined the mechanism of this activity. As iron chelation plays a role in the anti-proliferative activity of the DpT analogues [7, 8, 27], it was of interest to examine the effect of these compounds on heme-containing proteins, particularly those that could play an integral role in cellular metabolism.

Both cytoglobin (Cygb) and neuroglobin (Ngb) are intracellular globins (belonging to the same family as hemoglobin and myoglobin) containing the crucial heme prosthetic group that contains iron [51, 52]. These heme-containing globins have been reported to facilitate the diffusion of oxygen in tissues and also act as oxygen sensors and radical scavengers [51, 52]. The overexpression of both these proteins is found in hypoxia or under oxidative stress [52]. The effects of chelators on Cygb and Ngb in non-tumorigenic, immortalized cells relative to neuroblastoma cells remains unknown, and it was considered important to assess the effects of DpC and Dp44mT on these proteins. Indeed, their iron-containing heme groups could be indirectly affected by chelation of key intracellular iron pools in neoplastic cells [7, 8, 27], which may result in inhibition of protein function.

**Fig. 6:**
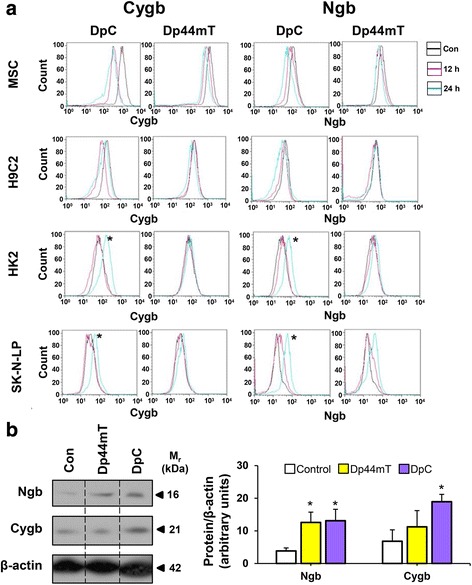
**a** Incubation of DpC (25 μM) with SK-N-LP neuroblastoma cells and HK2 non-tumorigenic, immortalized kidney cells induces Cygb and Ngb expression after a 24-h incubation. In these studies, non-tumorigenic, immortalized cell lines (i.e., MSC, H9c2, or HK2), or neoplastic, neuroblastoma (SK-N-LP) cells, were incubated for either 0, 12 or 24 h/37 °C with either control medium (*Con*; no agent added), Dp44mT (25 μM), or DpC (25 μM) and then flow cytometric analysis performed using Flow Jo 8.8.2. *Black*, *red*, and *blue lines* represent the control, or the cells treated with the iron chelators for 12 and 24 h, respectively. Results shown are typical experiments of three performed. **b** Western blot analysis of Cygb and Ngb expression in SK-N-LP cells following incubation with control media (*Con*), Dp44mT (25 μM), or DpC (25 μM) for 24 h/37 °C. The bands presented in the blots are representative of three repeats and the lanes have been cropped from raw data images containing all three repeats (raw data shown in Additional File 1) for clarity (lanes separated by *dotted lines*). Densitometry data in (**b**) are presented as the mean ± SEM (*n* = 3). *p < 0.05 by an unpaired two-tailed Student’s *t* test in triplicate experiments

Interestingly, flow cytometric analysis demonstrated that DpC significantly (*p* < 0.05) upregulated Cygb and Ngb expression in HK2 kidney cells and SK-N-LP neuroblastoma cells after an incubation with DpC (25 μM) for 24 h/37 °C (Fig. 6a). A less marked increase in Ngb expression was also observed in both HK2 and SK-N-LP cells after a 12 h/37 °C incubation (Fig. 6a). In contrast, Cygb and Ngb expression slightly decreased in MSC and H9C2 cells after incubation with DpC. However, unlike DpC, Dp44mT failed to significantly induce similar alterations in Cygb and Ngb expression in all cell lines tested, apart from a slight, but not significant (*p* > 0.05) increase in Ngb in the SK-N-LP cells (Fig. 6a). These results were also reflected in western blot studies using SK-N-LP cells, where DpC mediated a significant (*p* < 0.05) increase in both Cygb and Ngb expression levels, while Dp44mT only significantly (*p* < 0.05) affected Ngb expression (Fig. 6b). These observations suggested a difference in the mechanism of action of these two agents despite their similar structures (Fig. 1b, c).

### Effect of DpC and Dp44mT on key molecular pathways in neuroblastoma

**Fig. 7:**
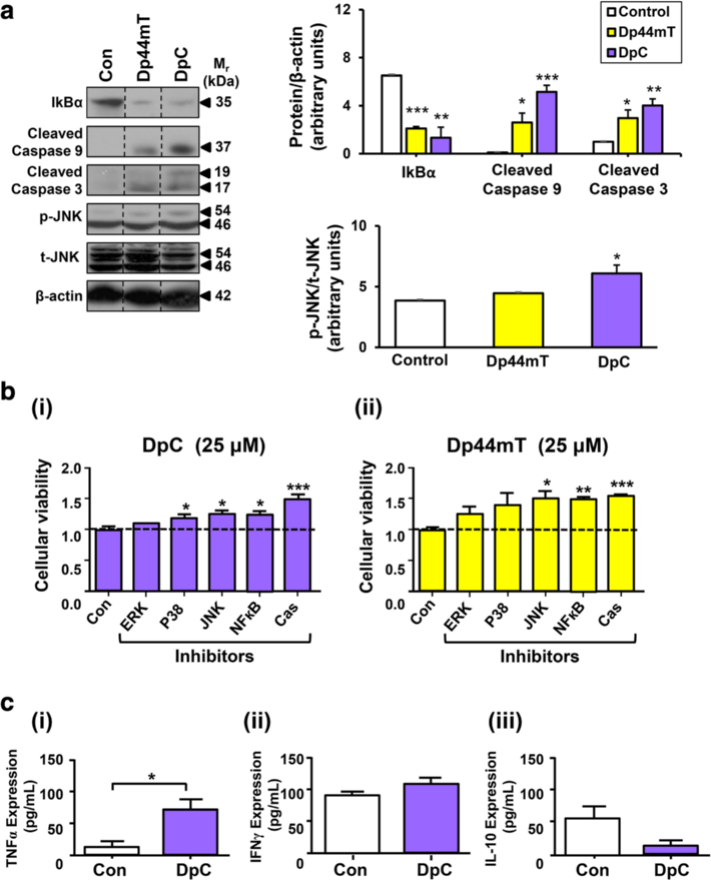
Molecular mechanisms involved in the DpC-mediated anti-neuroblastoma activity. **a** Western analysis demonstrating that incubation of SK-N-LP neuroblastoma cells with either Dp44mT or DpC (25 μM) for 24 h/37 °C significantly: (*i*) reduced IĸBα levels, (*ii*) increased cleaved caspase 9 levels, and (*iii*) increased cleaved caspase 3 levels. DpC also significantly increased the phosphorylated JNK (p-JNK)/total JNK (t-JNK) ratio. Western blotting was performed as described in the “Methods.” The bands presented are representative of three repeats and have been cropped from raw data images containing all three repeats (shown in Additional file 1) for clarity (blots separated by *dotted lines*). **b** The cytotoxic effects of (*i*) DpC and (*ii*) Dp44mT on SK-N-LP cells are significantly reduced upon inhibition of the JNK, NF-ĸB or caspase (Cas) pathways, while inhibition of p38 signaling only reduced DpC cytotoxicity. SK-N-LP cells were pre-incubated for 2 h/37 °C with ERK1, p38, JNK, NF-ĸB, or Cas inhibitors prior to incubation with either DpC (25 μM) or Dp44mT (25 μM) in the presence or absence of these inhibitors for 24 h/37 °C. Cell viability was assessed using the XTT assay, as described in the “Methods *.*” **c** Significantly higher levels of secreted (*i*) TNFα were detected in the xenografts of the DpC-treated mice (*p* < 0.05), while no significant changes in (*ii*) IFNγ or (*iii*) IL-10 secretion were detected. The levels of these cytokines were quantified via the ELISA assay, as described in the “Methods *.*” Data in graphs is presented as the mean ± SEM (*n* = 3). *p < 0.05; **p < 0.01; ***p < 0.001, as determined by an unpaired Student’s two-tailed *t* test

Considering that DpC and Dp44mT promote apoptosis in neuroblastoma cells (Figs. 3 and 5), further studies assessed the potential mechanisms involved in this effect by examining the key molecular pathways that initiate apoptosis in neuroblastoma cells, including the NF-ĸB and MAPK signaling cascades. Notably, both NF-ĸB and MAPK pathways, via their activation of p38 and JNK, lead to transcription of genes that promote apoptosis, namely TNFα, c-Jun, AP-1, cytochrome *c*, etc. [53, 54]. Further, these pathways also promote cleavage of caspase 8 and 9, which ultimately leads to caspase 3 cleavage and apoptosis [55]. Hence, western blot studies assessed the effects of DpC and Dp44mT on the expression of a key inhibitor of the NF-ĸB pathway, namely IĸBα [56], as well as the major regulator of MAPK signaling, that is JNK, and down-stream targets of these signaling pathways, including cleaved caspase 3 and 9 (Fig. 7).

Examining the NF-ĸB pathway, a significant (*p* < 0.01–0.001) decrease in IĸBα expression was observed after incubation with Dp44mT or DpC (Fig. 7a). Considering that IkBα inhibits NF-ĸB nuclear localization and function [56], a decrease in IĸBα expression will enable NF-ĸB activation. Further, a significant (*p* < 0.05–0.001) increase in cleaved caspase 3 and 9 was observed upon incubation with either DpC or Dp44mT (Fig. 7a). DpC also significantly (*p* < 0.05) increased the phosphorylated JNK/total JNK ratio, while having no significant (*p* > 0.05) effect on total JNK levels (Fig. 7a). These results indicate that Dp44mT may activate the NF-ĸB pathway and also the cleavage of caspase 3 and 9, while DpC activates both the NF-ĸB and MAPK pathways to promote apoptosis.

Considering the western results in Fig. 7a and to further investigate the role of the MAPK/NF-ĸB/caspase signaling pathway in the anti-proliferative activity observed with DpC, selective inhibitors of these pathways were utilized to assess the mechanism of the cytotoxicity of DpC (25 μM) or Dp44mT (25 μM; Fig. 7b). In these studies, a 2 h/37 °C pre-incubation of SK-N-LP neuroblastoma cells with p38, JNK, NF-ĸB, and caspase inhibitors prior to a 24 h/37 °C incubation with DpC and the inhibitors could slightly, but significantly (*p* < 0.001-0.05), reduce the cytotoxicity of DpC, while the ERK inhibitor did not have any significant (*p* > 0.05) effect (Fig. 7b). Similarly, the JNK, NF-ĸB, and caspase inhibitors could slightly and significantly (*p* < 0.001–0.05) decrease the cytotoxicity of Dp44mT, while the ERK and p38 inhibitors did not have a significant (*p* > 0.05) effect (Fig. 7b). For both DpC and Dp44mT, the caspase inhibitor was the most effective at inhibiting their cytotoxicity, suggesting the important role of caspases in DpC/Dp44mT-mediated apoptosis.

To further investigate the mechanism of action of DpC, in vivo studies were performed to assess its effects on TNFα, IFNγ, and IL-10 levels, as these are downstream targets of the MAPK/NF-ĸB/caspase signaling pathways [56, 57]. Considering the activation of these pathways in vitro in neuroblastoma cells by DpC (Fig. 7a, b), they could also be potentially activated by DpC in vivo. Interestingly, significantly (*p* < 0.05) higher TNFα levels were detected by ELISA assays in SK-N-LP tumor xenografts of the DpC-treated group (Fig. 7 Ci). Further, IFNγ and IL-10 were slightly increased or decreased in these xenografts, respectively, although these effects were not significant (*p* > 0.05) (Fig. 7Cii, iii).

## Discussion

The importance of the DpT series of analogues as new anti-cancer therapeutics is demonstrated by (1) their broad and selective anti-tumor activity [7, 8, 26, 27], (2) their ability to inhibit metastasis via up-regulation of NDRG1 or 2 [22–24], and (3) the efficacy of these compounds to overcome Pgp-mediated drug resistance [10, 12, 13]. In fact, in early 2016, DpC entered multi-center clinical trials for the treatment of advanced and resistant tumors (NCT02688101).

Considering the marked anti-tumor activity of the DpT analogues, their activity and mechanism of action was examined against the belligerent childhood tumor, neuroblastoma, in vitro and in vivo. The current studies have demonstrated in vitro that the commercially available chelator, L1, was markedly less effective than Dp44mT, and particularly DpC, in terms of its activity against neuroblastoma cells. This is probably because L1 does not form cytotoxic redox-active metal complexes upon saturation of its coordination sphere with iron (i.e., (L1)_3_Fe^III^), since its iron ligating sites are “hard” oxygen donors (Fig. 1d) which prevents redox cycling [58, 59]. This is in contrast to both Dp44mT and DpC, where “soft” N and S donors (Fig. 1b, c) in the coordination sphere enable the generation of redox-active metal complexes [8, 13, 42] that play an important role in the induction of apoptosis [9–12]. Hence, for L1, its major mechanism of action is confined to essential metal-binding and depletion that results in the inhibition of proliferation (a “single punch”), while Dp44mT and DpC act via binding essential metals and then redox cycling to generate a “double punch” to inhibit tumor growth [1, 8, 13, 42].

Importantly, in terms of the selectivity of these agents, a therapeutic window was observed in vitro at low concentrations where DpC and Dp44mT showed no anti-proliferative activity against the panel of non-tumorigenic, immortalized cells (i.e., MSC, H9C2, MIHA, and HK2), but did inhibit the neoplastic, neuroblastoma cell line, SK-N-LP (Fig. 2b). This was in good agreement with previous studies in other tumor cell types in vitro, where selective anti-cancer activity and a therapeutic window was observed for Dp44mT and DpC [7, 8, 26, 27]. Moreover, the marked anti-tumor activity of DpC was independent of *MYCN* amplification, which is a key oncogene and prognostic indicator in neuroblastoma [45–47]. It was also of interest that Dp44mT demonstrated relatively higher efficacy against *MYCN* amplified cell lines relative to neuroblastoma cells without *MYCN* amplification.

The studies demonstrating the marked and selective anti-neuroblastoma efficacy of DpC in vitro were confirmed in vivo, where this agent decreased neuroblastoma growth without major toxicology. Furthermore, mouse body weights in the DpC-treated group did not show a distinct decline relative to the vehicle control in concordance with prior reports [26, 27], although there was a slight decrease in weight gain in the DpC-treated group. However, in contrast to a previous investigation using a less intensive dosing schedule [26], DpC was shown to induce lung inflammation (Fig. 5d). This effect may be due to the more intensive treatment regimen implemented herein (i.e., 7 days/week vs. 5 days/week with 2 days rest used previously) and indicates that careful titration of the dose is required to ensure appropriate anti-cancer activity without toxic effects.

In terms of the decreased neuroblastoma growth observed in vivo, it is notable that the effect of DpC on the tumor was not merely cytostatic, but cytotoxic, as there was significantly elevated caspase 3 and Annexin V (+)/PI (+) staining in the tumor after DpC treatment, indicating increased apoptosis. Such cytotoxicity within the neuroblastoma tumor was important to demonstrate, as the induction of cytostasis is of little benefit to patients, particularly when drug administration is stopped, since it leads to tumor rebound.

Considering the mechanism of action of the DpT analogues and the role of iron in their activity [7, 8, 27], it was of interest to examine the effect of the agents on heme-containing proteins, particularly those that play a role in metabolism. While chelators do not directly remove iron from heme itself, they could affect iron trafficking pathways subsequent to its incorporation into heme. Both Cygb and Ngb are intracellular heme-containing proteins of the globin family that play roles in oxygen metabolism and appear to act as reactive oxygen species scavengers [60–63]. DpC significantly upregulated Cygb and Ngb expression in HK2 kidney and SK-N-LP neuroblastoma cells (Fig 6a, b), while Dp44mT only increased Ngb levels (Fig. 6a, b). There has been little work to assess the role of iron-depletion on the expression of either Cygb or Ngb, but a study in rats demonstrated that a low iron diet reduced Ngb levels [64]. Considering this, it can be suggested that the ability of DpC and Dp44mT to chelate iron is not the cause of the increase in Ngb expression (Fig. 6). In contrast, since Cygb expression occurs under oxidative stress [65], it can be speculated that the potent oxidative stress induced by DpC metal complexes [13] may be involved in increasing Cygb and Ngb expression. Thus, the increase in Ngb and Cygb levels after incubation with DpC may represent a protective response. However, it is unclear why Dp44mT, which is also redox active and has a similar mechanism of cytotoxic activity to DpC [9–13, 42], did not increase Cygb expression.

As part of their complex mechanism of action, previous studies have indicated that Dp44mT and DpC have marked effects on multiple signaling pathways in other tumor types [15–21]. Significantly, the current study also demonstrated that DpC increased the levels of phosphorylated JNK and cleaved caspase 3 and 9, while it decreased IkBα expression (an inhibitory factor of NF-ĸB signaling; [56]) in neuroblastoma cells in vitro. In contrast, Dp44mT was less effective and only mediated an increase in cleaved caspase 3 and 9 and a decrease in IkBα expression, while not significantly affecting phosphorylated JNK.

**Fig. 8:**
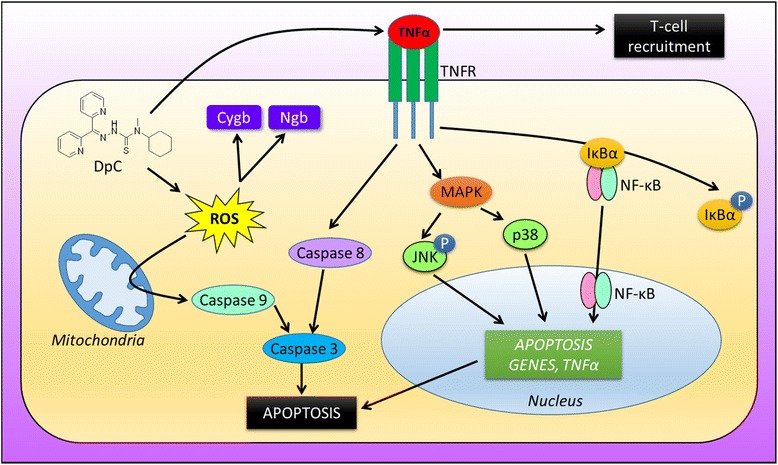
Overview of the potential mechanisms involved in the DpC-mediated effects on neuroblastoma. DpC increases TNFα expression in neuroblastoma cells, which may (1) activate cytotoxic T cells to destroy tumor cells and/or (2) acts on the TNFα receptor (*TNFR*) to activate down-stream signaling pathways. These include the MAPK/p38/JNK and NF-ĸB signaling cascades, which lead to nuclear transcription of numerous genes, including those that induce apoptosis, as well as cytokines such as TNFα. Activation of TNFR also promotes cleavage of caspase 8, leading to caspase 3 cleavage and subsequent apoptosis. Moreover, DpC is also highly redox active, resulting in the production of reactive oxygen species (ROS) [13]. The generation of ROS triggers the release of cytochrome *c* from mitochondria [8], leading to cleavage of caspase 9, which then also cleaves caspase 3, leading to apoptosis. Further, the increased ROS also leads to upregulation of neuroglobin (*Ngb*) and cytoglobin (*Cygb*) expression as both of these proteins respond to oxidative stress. Together, these molecular effects, which promote apoptosis, could contribute to the anti-cancer activity of DpC in neuroblastoma

Importantly, the ability of DpC to increase TNFα expression in neuroblastoma tumors in vivo may potentially contribute to these pro-apoptotic signaling effects shown in vitro, as TNFα binds to the TNFα receptor (TNFR) to activate the MAPK/p38/JNK and NF-ĸB signaling cascades, which lead to nuclear transcription of genes that induce apoptosis (Fig. 8) [66]. Further, the redox activity of DpC [13, 67] may also promote the release of cytochrome *c* from mitochondria (as found for Dp44mT; [8]), which mediates the cleavage of caspase 9, leading to apoptosis [8] (Fig. 8).

Notably, aberrations in NF-ĸB/IĸBα and MAPK signaling are closely linked to cancer development [68, 69] and are involved in integrating oncogenic signaling [33, 70]. Studies in vitro with inhibitors of p38, JNK, NF-ĸB, and caspases suggested their involvement in terms of the mechanism of action of DpC against neuroblastoma (Fig. 8). However, while these inhibitors did reduce the anti-proliferative efficacy of DpC, they were not markedly effective and did not totally inhibit its activity. This observation suggests the mechanism of action of DpC in neuroblastoma is via their activity on multiple molecular targets (Fig. 8) and underlines the importance of polypharmacology in their marked activity [28].

Finally, considering the potential effects of DpC on the immune system, it is of note that TNFα levels were significantly increased in vivo in neuroblastoma xenografts post-DpC treatment (Fig. 7 Ci). This finding was associated with a slight, but not significant, increase in IFNγ and decrease in IL-10. Notably, TNFα, together with IFNγ, plays an important role in initiating the immune response by activating tumor-specific cytotoxic T cells [66]. Hence, the ability of DpC to increase TNFα in tumors could promote the endogenous immune response to mediate immune cell infiltration of the cancer. Such an immune response could also be potentially implicated in the ability of DpC to inhibit neuroblastoma growth in vivo.

## Conclusions

In conclusion, DpC demonstrated a potent cytotoxic profile against neuroblastoma cells with or without *MYCN* amplification in vitro and was demonstrated to effectively inhibit orthotopic neuroblastoma xenograft growth in vivo without causing marked toxicity. In terms of its molecular mechanism of action against neuroblastoma tumors, DpC significantly increased levels of phosphorylated JNK, neuroglobin, cytoglobin, and cleaved caspase 3 and 9, while simultaneously decreasing inhibitory IĸBα levels in vitro (Fig. 8). Together, these results suggest that DpC may have a promising role in neuroblastoma treatment.

## Additional file

Additional file 1: Figure S1. Raw Western blot images of Ngb, Cygb, IĸBα, cleaved caspase 9 and 3, phosphorylated JNK (p-JNK) and total JNK (t-JNK) expression relative to the loading control, β-actin, in human SK-N-LP neuroblastoma cells following incubation with DpC (25 μM; Lanes 1-3), control medium (Lanes 4-6) or Dp44mT (25 μM; Lanes 7-9) for 24 h/37oC. Triplicates represent protein lysates obtained from 3 separate experiments. (PDF 108 kb)

## Abbreviations

3-AP/Triapine: 3-Aminopyridine-2-carboxaldehyde thiosemicarbazone
Cygb: Cytoglobin
DCF: Dichlorofluorescein
DFO: Desferrioxamine
DMSO: Dimethyl sulfoxide
DpC: Di-2-pyridylketone 4-cyclohexyl-4-methyl-3-thiosemicarbazone
Dp44mT: Di-2-pyridylketone 4,4-dimethyl-3-thiosemicarbazone
ELISA: Enzyme-linked immunosorbent assay
H & E: Hematoxylin-eosin
IHC: Immunohistochemistry
IFNγ: Interferon gamma
IL-10: Interleukin 10
IVIS 100: In vivo imaging system Xenogen 100
IkBα: Nuclear factor of kappa light polypeptide gene enhancer in B-cells inhibitor alpha
L1: Deferiprone
MSC: Mesenchymal stem cell
Ngb: Neuroglobin
NDRG1: N-myc downstream regulated gene-1
NF-kB: Nuclear factor kappa B
PBS: Phosphate-buffered saline
ROS: Reactive oxygen species
TNFα: Tumor necrosis factor alpha
TNFR: Tumor necrosis factor receptor
