# Supplementary material for: The novel thiosemicarbazone, di-2-pyridylketone 4-cyclohexyl-4-methyl-3-thiosemicarbazone (DpC), inhibits neuroblastoma growth in vitro and in vivo via multiple mechanisms
Source: J Hematol Oncol. 2016 Sep 27;9:98. doi: 10.1186/s13045-016-0330-x (PMC5039880; doi:10.1186/s13045-016-0330-x)
Supplement: Additional file 1: Figure S1. — Raw Western blot images of Ngb, Cygb, IĸBα, cleaved caspase 9 and 3, phosphorylated JNK (p-JNK) and total JNK (t-JNK) expression relative to the loading control, β-actin, in human SK-N-LP neuroblastoma cells following incubation with DpC (25 μM; Lanes 1-3), control medium (Lanes 4-6) or Dp44mT (25 μM; Lanes 7-9) for 24 h/37oC. Triplicates represent protein lysates obtained from 3 separate experiments. (PDF 108 kb) [file 13045_2016_330_MOESM1_ESM.pdf]

Supplementary Data

The Novel Thiosemicarbazone, Di-2-pyridylketone 4-Cyclohexyl-4-methyl-3-thiosemicarbazone (DpC), Inhibits Neuroblastoma Growth *In Vitro* and *In Vivo via* Multiple Mechanisms

Zhu-Ling Guo[1,2], Des R. Richardson[3*], Danuta S. Kalinowski[3], Zaklina Kovacevic[3], Kian Cheng Tan-Un[4, 5] and Godfrey Chi-Fung Chan[1*]

[1]*Departments of Paediatrics & Adolescent Medicine, Li Ka Shing Faculty of Medicine, the University of Hong Kong, Hong Kong SAR, PR China.*

[2]*School of Stomatology, Hainan Medical University, Hainan, PR China.*

[3]*Molecular Pharmacology and Pathology Program, Department of Pathology, University of Sydney, Sydney, New South Wales, Australia.*

[4]*School of Biological Sciences, The University of Hong Kong, Hong Kong SAR, PR China.*

[5]*School of Professional and Continuing Education, The University of Hong Kong, Hong Kong SAR, PR China.*

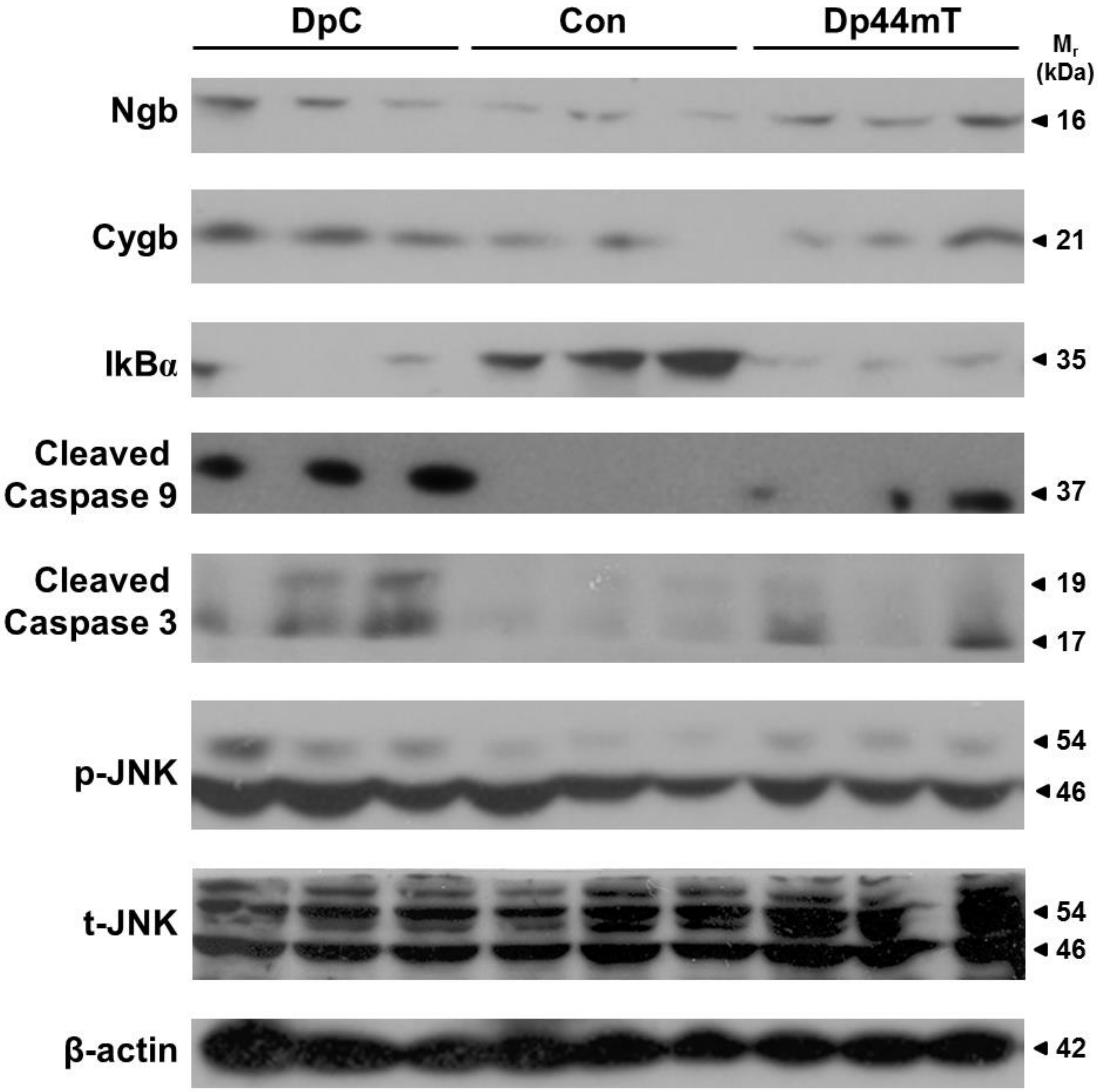

Figure S1. Raw Western blot images of Ngb, Cygb, IκBα, cleaved caspase 9 and 3, phosphorylated JNK (p-JNK) and total JNK (t-JNK) expression relative to the loading control, β-actin, in human SK-N-LP neuroblastoma cells following incubation with DpC (25 µM; Lanes 1-3), control medium (Lanes 4-6) or Dp44mT (25 µM; Lanes 7-9) for 24 h/37°C. Triplicates represent protein lysates obtained from 3 separate experiments.
